# First Report of Epimeletic and Acoustic Behavior in Mediterranean Common Bottlenose Dolphins (*Tursiops truncatus*) Carrying Dead Calves

**DOI:** 10.3390/biology11020337

**Published:** 2022-02-21

**Authors:** Giulia Pedrazzi, Giancarlo Giacomini, Daniela Silvia Pace

**Affiliations:** Department of Environmental Biology, Sapienza University of Rome, 00185 Rome, Italy; g.pedrazzi06@gmail.com (G.P.); giancgiacomini@gmail.com (G.G.)

**Keywords:** epimeletic behavior, bottlenose dolphins, *Tursiops truncatus*, dead calf, acoustic behavior, Tiber River, Mediterranean Sea

## Abstract

**Simple Summary:**

Death-related behaviors have been often observed in cetaceans, frequently with a female caregiver (presumed mother) supporting a dead calf by carrying, lifting, or sinking it, generally accompanied by other escorting individuals. However, how cetaceans experience death and if their behavior could be compared to human grieving is still largely unknown, as well as their acoustic production in such contexts. This report describes two cases of an adult common bottlenose dolphin (*Tursiops truncatus*) supporting a dead newborn and associated acoustic behavior in the Tiber River estuary area (Rome, Mediterranean Sea, Italy). In both cases, a main supporter (putative mother) was observed interacting with the carcass of a newborn by lifting (case 1) and sinking it (case 2), always escorted by another adult individual. Several vocalizations were recorded, including a signature whistle (a tonal sound with a repeated, stereotyped, individual-specific frequency modulation pattern) in the first case, likely emitted by the putative mother to seek aid from other individuals. This result appears reasonable since bottlenose dolphins are a highly vocal species, that communicates mainly through acoustic signals. These observations confirm the occurrence of death-related supportive behavior in bottlenose dolphins and report a preliminary description of associated vocalizations, providing additional information on this largely unknown topic.

**Abstract:**

Epimeletic behavior toward dead calves has been frequently reported in cetaceans, mostly with females (presumed mothers) showing supportive behaviors such as carrying, lifting, or sinking, often assisted by “escort” individuals. However, information on acoustic production in such contexts is scarce. This report describes two observations of epimeletic behavior toward dead newborns in bottlenose dolphins and associated acoustic production. Data were collected at the Tiber River estuary (Rome, Mediterranean Sea, Italy) with one hydrophone for passive acoustic monitoring and two digital cameras. In both cases, an individual (presumed mother) acting as the main supporter and directly interacting with the carcass by lifting it (case 1) or sinking it (case 2) was observed. Another adult individual (escort) was present in both encounters showing standing-by and excitement behaviors (case 1) and supportive behavior (case 2). In both encounters, whistles, pulsed sounds, and bray-call elements were recorded. The consistent vocal activity observed likely conveyed context-specific information. A signature whistle in the first encounter was also recorded, likely emitted by the putative mother as a distress call. This report confirms the occurrence of epimeletic behavior in bottlenose dolphins and reports a preliminary description of the acoustic production when a dead calf is involved, providing additional information on this largely unknown topic.

## 1. Introduction

Behavioral responses to dead conspecifics have long been thought to be a unique feature of the human species. However, observations of individuals showing supportive behavior toward dead or dying conspecific in the wild have been reported for several terrestrial and marine mammal species, including chimpanzees [1], giraffes [2], elephants [3], and cetaceans [4]. Death-related behaviors can be considered a subtype of epimeletic behavior, which occurs when a healthy individual (supporter) gives attention to or takes care of another distressed, injured, dying or, dead individual (receiver) [5]. Caldwell and Caldwell [6] distinguished two different categories of epimeletic behavior: nurturant (supportive behavior directed toward younger individuals) and succorant (support aimed at adults). In cetaceans, nurturant behavior is the most reported, frequently directed toward dead newborns [7]. When a calf is involved, the caregiver appears to be a female (presumably the mother) in most of the cases in which its sex is known [5]. The supporter may often be accompanied by other individuals, called “escorts”, who either directly assist the caregiver interacting with the receiver or remain close and swim by the pair [5,8]. Post-mortem supportive behavior in cetaceans generally includes stereotyped behaviors that can be divided into three main groups: (1) standing-by, with individuals remaining close to the carcass without directly interacting with it, (2) excitement, with participants showing behaviors typical of arousal states such as erratic swimming, and (3) supportive behavior, with individual interacting directly with the carcass, typically lifting, sinking or carrying it with the melon, the rostrum, or the edge of the dorsal fin [9,10]. 

Among cetaceans, the common bottlenose dolphin (*Tursiops truncatus*) is one of the species most frequently reported to exhibit epimeletic behavior [4,5,11]. Consistently with other cetacean species, death-related behaviors are mainly directed from an adult female toward a younger individual (presumably its offspring) and they include carrying, mouthing, lifting, and diving [4]. Bottlenose dolphins are a highly vocal species with a complex vocal repertoire [12]. They show strong vocal plasticity [13] and are known to adapt the emission rates and the acoustic features of their vocalizations depending on the contexts and emotional state [14,15,16]. However, despite the consistent number of reports on epimeletic behavior for this species, still little is known about their acoustic production in such contexts, and little evidence has been reported. The only available description of acoustic production during epimeletic behavior in bottlenose dolphins is reported by Kuzcaj et al. [17] that characterized the whistles emitted during a case of succorant behavior directed toward a distressed conspecific. They found out that signature whistles were emitted as distress calls, with higher emission rates and higher intensity to seek aid from other individuals [17]. Few other reports provide a general description of the acoustic production in epimeletic-related contexts. The study from Perrtree et al. [18] reported higher emission rates of the signature whistle emitted by a mother during an infanticide attempt in bottlenose dolphins, possibly expressing context-related information. The one from Cheng et al. [9] characterized the whistles produced by an adult Indo-pacific humpback dolphin (*Sousa chinensis*) during nurturant behavior directed toward a dead calf. They reported a longer duration and higher number of inflection points in whistles emitted during nurturant behavior than in whistles produced in other contexts and hypothesized that these variations may convey information on the emotional state of the emitter [9]. Finally, [19] reported consistent whistles emission during a case of succorant behavior in bottlenose dolphins, with possible distress function.

Here, two episodes of nurturant behavior toward dead calves observed in 2021 in the population of bottlenose dolphins inhabiting the Tiber River estuary area (Rome, Italy, Mediterranean Sea), and associated acoustic production, are reported. This is the first detailed evidence of epimeletic behavior involving a dead calf in this population, although another case was observed by a sea-user in 2015 [20]. The final aim of this report is to provide additional information on death-related behaviors in such a species, moving toward a deeper comprehension of this still largely unknown behavioral context.

## 2. Materials and Methods

### 2.1. Study Area

**Figure 1:**
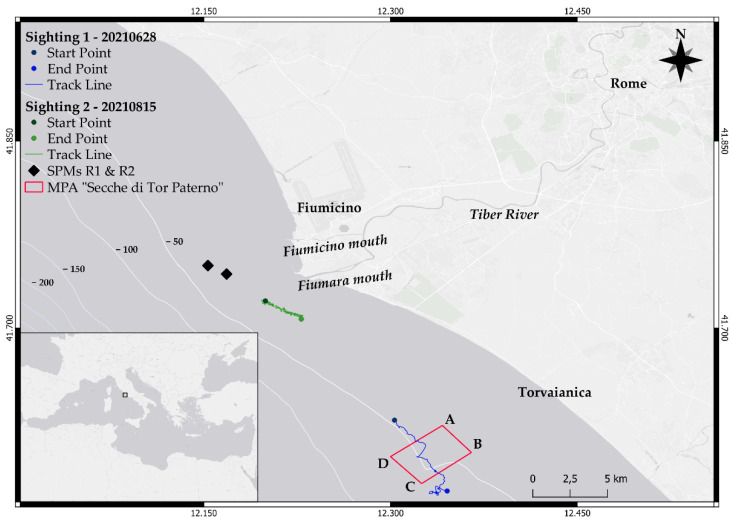
Map of the study site. The red rectangle represents the marine protected area “Secche di Tor Paterno” while the two diamonds represent the Single Point Moorings R1 and R2. The map also shows start and ending points (dots) and tracks (lines) of the first (blue) and the second (green) sighting.

The study site is approximately 1300 km^2^ and is located in the central Mediterranean Sea (Tyrrhenian Sea, Rome, Italy) (Figure 1). It includes the Tiber River estuary, which flows into the sea through the two mouths of Fiumara Grande (natural mouth) and Fiumicino (artificial mouth).

The area is rich in habitats and environmental conditions, and is characterized by a flourishing marine community that developed partly because of the large amount of organic material transported by the river [21]. However, crossing the city of Rome, the Tiber also contributes to enhancing pollution levels, bringing to the sea big quantities of pollutants, heavy metal and waste [21]. The study site also includes the “Secche di Tor Paterno” marine protected area and two single point moorings (SPMs, called R1 and R2) for the reception of crude oil and the supply of petroleum products (structures known to attract bottlenose dolphins [22]). 

The area is well recognized for the presence of bottlenose dolphins [23], with the regular observation of mother-calf pairs and foraging behavior, thus appearing a suitable calving and feeding ground for the species [20,21]. To date, 347 unique individuals have been photo identified in the area, showing different patterns of site fidelity [21]. A resident group of 42 individuals with low dispersion level has been identified, half of which are classified as females [21]. The remaining animals have been classified as part-time residents (*n* = 73), and transients (*n* = 232), showing lower levels of site fidelity and moving in and out of the study area [21]. No information is currently available on males, as the dolphins’ genital area was observed only occasionally, not allowing us to use this criterion to classify individuals as putative males.

The area is largely exposed to anthropogenic disturbance. Indeed, the two ports of Fiumicino (fishing port) and Ostia (touristic port) generate intense vessel traffic, in addition to several shipyards located on the riverbank. The site is also relevant for commercial fisheries (artisanal and trawlers) and interactions between dolphins and fishing gears have been frequently reported, likely because their target species greatly overlap. For a more detailed description of the study site, see Pace et al. [21].

### 2.2. Data Collection

The two cases of epimeletic behavior were recorded as part of an intensive research program on common bottlenose dolphins run by the Department of Environmental Biology (DBA) of “La Sapienza” University of Rome since 2017 in the Roman seas. The collection of acoustic and visual data was carried out during daily surveys using a sailing vessel, Beneteau Oceanis 41.1 powered by a 55 hp Volvo diesel engine, in suitable weather conditions (i.e., sea state < 3 Douglas, wind force Beaufort < 3, no rain, no fog), at a steady speed of 4–6 knots, following non-systematic haphazard sampling procedure [24]. Acoustic data were collected using one towed hydrophone Aquarian Audio (Anacortes, WA, USA, model H1c-2018 provided by Nauta srl; sensitivity −199 dB re 1 V/μPa; flat frequency response from 20 Hz to 4 kHz ± 4 dB with a bandwidth between <0.1 to >100 kHz) and one digital sound card Roland Quad Capture.

When a sighting occurred, the survey effort was suspended, and the route changed to approach the dolphins, remaining at a safe distance to not interfere with their behavior (>100 m). During the encounter, GPS location, time, direction, group size and composition (based on the classification reported in [21]) and predominant behavior (i.e., the behavioral state in which more than half of the individuals within the group are involved; [25]) were recorded. All occurring behavioral states and events were logged following *ad libitum* and *all occurrences* sampling methods [26]. Photographs of dorsal fins were collected using Canon digital 5D and 6D cameras and 100–400 mm f/4.5–5.6 L zoom lens for photo-identification (details on photo-identification procedure in Pace et al. [21] and Mariani et al. [27]).

### 2.3. Data Analysis

Behavioral records and photographs were examined to recognize patterns occurring during the sightings and identify individuals through their natural markings.

Recordings were analyzed using Raven Pro 1.6 [28] to distinguish different vocalizations emitted during the encounters. The visual inspection of the spectrograms was used to classify each call type in: whistle (tonal narrow-band sound, lasting at least 0.1 s, with part of the fundamental frequency above 3 kHz [29]), click train (highly directional broadband impulsive sound used for echolocation purposes [30]), burst pulses (pulsed sound with a repetition rate of 300 pulses per second and inter-click-interval shorter than 3 ms [30,31], and bray-call elements (gulp, grunt and squeak [32]). Different spectrogram parameters were used for each type of vocalization, as reported in Table 1. 

A quality score was assigned to each vocalization depending on the signal-to-noise ratio and only good- to high-quality whistles and pulsed sounds were further analyzed to extract specific acoustic parameters (Table 2). Bray-call elements were only reported as presence/absence. Whistle contours were categorized as Single Element (SE), Connected (CML), or Disconnected (DML) Multi-Loop, according to their loop structure [29]. Finally, considering their contour’s repetition pattern, each whistle was classified as Signature Whistle (SW), Repeatedly-Emitted Whistle Type (REWT), or Other Whistles (OW), following the SIGID method criteria [33]. The average and standard deviation for each extracted parameter was calculated using R 4.0.3 (www.r-project.org; accessed 22 December 2021).

## 3. Results

### 3.1. Sighting 1

**Figure 2:**
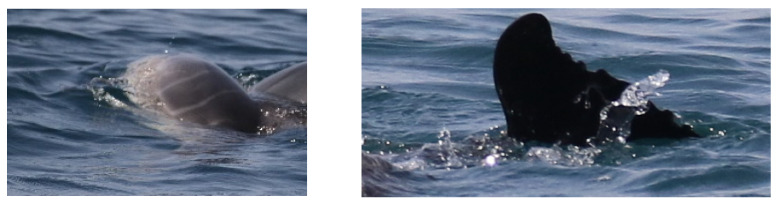
**Left** picture: the dead new-born with visible fetal folds. **Right** picture: Edge of the new-born tail visibly marked.

The first encounter occurred on 28/06/2021 and lasted approximately 3 h, from 09:19 UTC to 12:21 UTC. The route of the sighting is shown in Figure 1 (blue line and dots). Initially, a single adult bottlenose dolphin swimming slowly was spotted; when closer, two adults swimming around a fresh carcass of a dead younger individual were observed. The dead individual was defined as a newborn because of the presence of distinguishable fetal folds (Figure 2, left panel). It was likely recently dead as the belly was flat, and the body was not floating on the surface; the edge of the tail appeared marked (Figure 2, right panel). Both adults did not match any individual included in the photo-ID catalog of the population and their sex is unknown. Here, the main supporter (or putative mother) who was directly interacting with the carcass is reported as individual “A”, and the other who was swimming around the supporter-receiver pair as individual “B”. 

**Figure 3:**
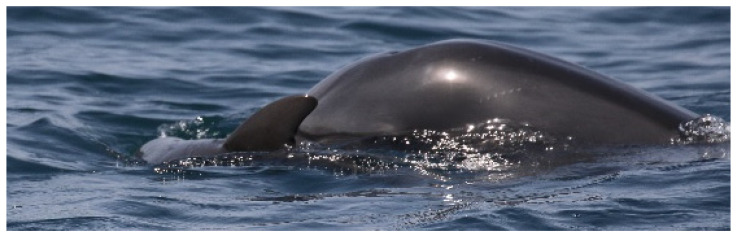
The putative mother (“A”) carrying the dead calf on her rostrum.

**Figure 4:**
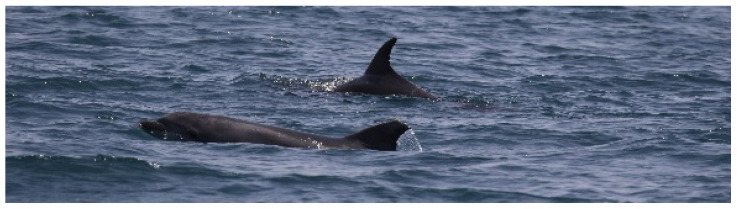
The individual “B” (**bottom** dolphin) swimming close to the putative mother (**upper** dolphin).

**Figure 5:**
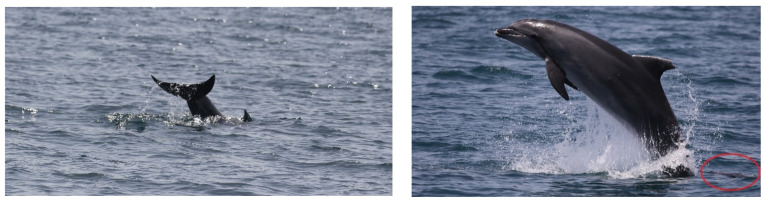
Tail slap (**left** picture) and leaping close to the carcass (red circle) exhibited by the escort individual “B” (**right** picture).

“A” spent most of the time carrying the carcass on its rostrum and trying lift it, bringing the dead body to the surface, and pushing it up with the rostrum repeatedly every time it sunk. (Figure 3). Considering the time that “A” spent in supportive behavior toward the dead calf, we presumed this animal to be the mother. The other individual “B” remained close during the entire sighting, swimming around the pair without directly providing aid most of the time (Figure 4) and showing several behaviors typical of arousal states such as tail-slapping (Figure 5, left picture) or leaping close to the carcass (Figure 5, right picture). During one of the leaps, the genital area of the animal briefly appeared, allowing us to presume this individual to be a putative male, although we did not manage to take any pictures to confirm it. 

The presumed mother continued with the supportive behavior until the end of the sighting (the research team decided to leave the animals after three hours spent in documenting the event). Since the putative mother was still strongly interacting with the dead calf when the research team left the animals, the carcass was not recovered, nor any attempt made to obtain it, to not disrupt her behavior.

Passive acoustic monitoring for the entire duration of the encounter was used to record the acoustic behavior of the animals. A total of 179 min in 19.wav files were collected, identifying 179 click trains, 2 burst pulses, and 70 whistles. Several acoustic sequences containing bray-call elements were also detected. Twenty-two out 70 identified whistles were good enough to extract the acoustic parameters and be further characterized. Based on the repetition of the same whistle contour and according to the SIGID criteria, 10 whistles were classified as a single signature whistle (SW_1) and the remaining as other whistles (OWs). 

**Figure 6:**
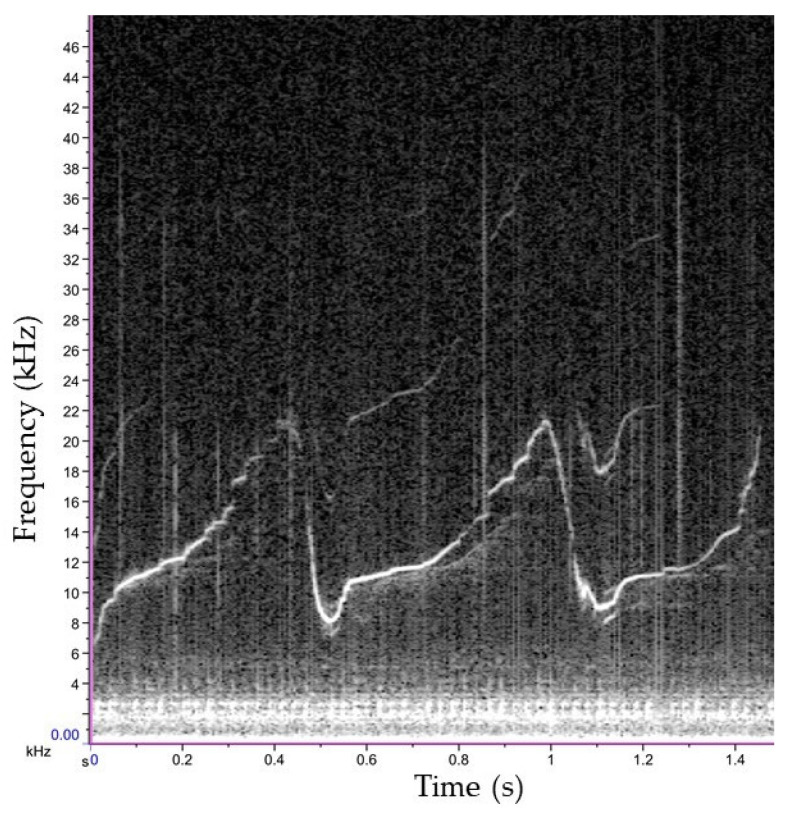
Signature Whistle (SW_01) identified in the recordings from the first encounter (Hamming window, size 2048, DFT 2048, Overlap 50 %, Hop size 1024).

The mean and standard deviation of the acoustic parameters extracted from OWs and SW_1 are reported in Table 3. All OWs presented a modulated contour (except one ascendant), with eight identified as Single Element (SE), and four Connected Multi-Loop (CML). The contour of the whistle recognized as SW_1 is reported in Figure 6. This signature whistle, which was consistently repeated during the sighting (41% of analyzed whistles), presented a modulated contour consisting of two connected loops with three inflection points, overtones and several steps that made the contour appear quavering.

About 70% of the identified whistles (including the SW) was identified in the same recording, between 11:36 UTC and 11:46 UTC, just before and during the coming of three other individuals. These animals were not close enough to be photographed and remained in the area for 10 min without closely approaching the trio (“A”, “B” and the carcass). 

### 3.2. Sighting 2

The second encounter occurred on 15 August 2021. It started at 06:49 UTC and lasted approximately 4 h, until 10:47 UTC, when the research team decided to end the sighting and leave the animals. The route followed during the sighting is reported in Figure 1 (green dots and line).

**Figure 7:**
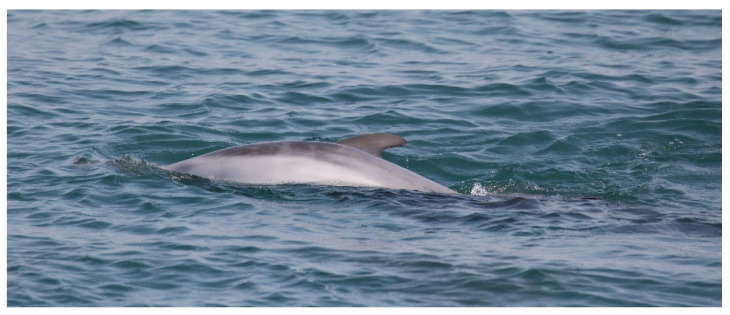
Carcass of the calf with the fetal folds still visible and inflated belly.

The group consisted of two adults and a dead younger individual. The main supporter was an adult resident female (UNIRM_142), known since 2018. The second adult who was escorting the main supporter was another adult resident female (UNIRM_025), known since 2017. Again, the dead individual was a calf (fetal folds still visible; Figure 7).

**Figure 8:**
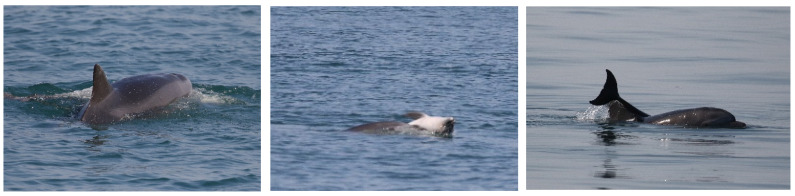
UNIRM_142 passing over the carcass (**left** picture), pushing it up with the rostrum (**middle** picture) and carrying it on the dorsal fin (**right** picture).

Different from the first case, the dead body showed an inflated belly and was floating on its back with stiff pectoral fins. The carcass condition suggested that death had occurred longer before than in the previous case. Because of the floating nature of the carcass, UNIRM_142 spent most of the time passing over the dead body to push it down into the water (Figure 8 left picture), occasionally pushing up the carcass with the rostrum (Figure 8 middle picture) or carrying it on the dorsal fin (Figure 8 right picture). 

**Figure 9:**
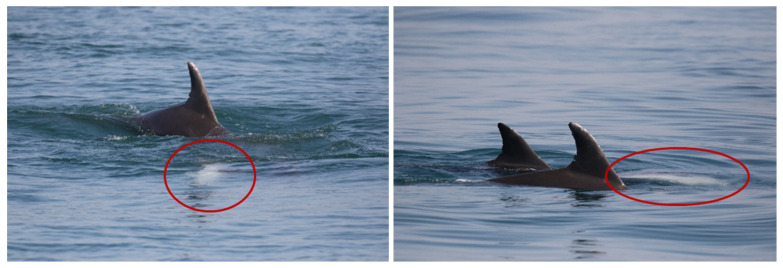
Left picture: UNIRM_025 passing over the carcass; right picture: UNIRM_025 (**right** dolphin) swimming close to UNIRM_142 (**left** dolphin). Red circles highlight the carcass.

The escorting individual UNIRM_025 directly participated in the supportive behavior, passing over the carcass (Figure 9 left picture). When not directly providing aid, UNIRM_025 remained close to the pair, swimming next to UNIRM_142 (Figure 9 right picture). This behavioral pattern continued until the end of the sighting (the research team decided to leave the animals after about four hours spent in documenting the event). Again, the carcass was not recovered, nor any attempt made to obtain it, to not disrupt the putative mother’s behavior, considering that both UNIRM_142 and UNIRM_025 were still fully interacting with the dead calf.

A total of 233 min in 24.wav files were collected, identifying 733 click trains, 4 burst pulses, 11 whistles and 3 bray-call elements (grunts). Unfortunately, in this case, good quality vocalizations to extract acoustic parameters were not present in the recordings.

## 4. Discussion

This report describes two different cases of epimeletic behavior by adult bottlenose dolphins toward dead calves in the Roman seas (Italy). Cases of nurturant behavior involving an adult (most frequently a female) and a dead calf (presumably its offspring) represent the majority of reports on epimeletic behavior in cetaceans [5,7,34]. In both encounters here reported it was not possible to assess the kinship of the main supporters, and it was also not possible to assess the sex in the first case, although we presumed them to be putative mothers considering the time and effort they spent in supporting the dead calves. Indeed, nurturant behavior has been proposed to derive from the disruption of the strong social bond that links a mother to its offspring [4] and from the interruption of the maternal cares [11]. This could be especially true for species in which females are the main caregivers of the offspring which is the case for bottlenose dolphins [10]. Mothers care for their offspring for the first 3–4 years of life (e.g., [35]) nursing, protecting, playing with, and maintaining proximity to their calves [36]. The loss of the calf may induce the mother to exhibit stereotyped behavior toward her dead calf for prolonged periods, even when the carcass is in an advanced state of decomposition [5]. Here the putative mothers supporting the dead calves spent hours without feeding, with potential negative effects on their fitness. However, making inferences on how long this behavior continued after the research team left the animals is difficult, since the duration may differ considerably from case to case [8,37]. 

The presence of other individuals has also been commonly documented during epimeletic behavior both in cetaceans [5,8,9] and in other mammals [1,38], likely suggesting that the death of a calf may affect an entire social group [8]. Here, the two escorts showed different behaviors: in the first case, the second adult “B”, which is presumed to be a male, remained close to the pair never providing aid. “B” interacted with the pair only on a few occasions, tail slapping or leaping close to the dead calf. Maintaining proximity could be interpreted as standing-by behavior as described by Caldwell et al. [6], with escort individuals swimming close to the main supporter without directly providing aid, while tail slaps and leaps could be interpreted as a form of excitement [6]. Tail slaps are also considered aggressive interactions, exhibited frequently as stress responses [39], and also observed during infanticide events [40], so they could be possibly explained as a reaction to the stressful context occurring or as an aggressive behavior exhibited by the putative male toward the presumed mother-calf pair. However, these hypotheses could not be confirmed since the occurrence of infanticide and the male’s relationship with the putative mother and the dead calf were not documented. On the other hand, leaps are known to be used as non-vocal communication cues and are often exhibited during social interactions [41], thus suggesting their potential role in communicating context-specific information. In the second case, the escort actively interacted with the carcass, providing direct support to the dead calf, and repeating the behavior exhibited by the putative mother. Nurturant supportive behavior between unrelated individuals has been reported in cetaceans, especially with females assisting unrelated calves [8,11,42], and can be considered as a form of altruism [9], likely deriving from the strong social bond between two individuals [11]. This could be the case for the second encounter here reported, considering that at least half of the resident individuals of this population are females that show high site fidelity and low dispersion [21]. These factors could be the basis for the development of strong associations between them [43], a phenomenon especially observed between females residing in estuarine regions [44,45] like the one here investigated.

The behaviors displayed by the putative mothers are consistent with other observations of epimeletic behaviors in bottlenose dolphins, which include carrying, lifting, and sinking the carcass [4]. These behaviors could be interpreted as an attempt to rescue an apparently inanimate individual [5], likely aimed at facilitating breathing or stimulating movement [17]. Lifting could also be seen as a transposition of the tendency of cetacean mothers to bring to the surface non-breathing newborns [46], and carrying as a form of protection [18]. Despite being energetically costly and apparently maladaptive for the supporter [5], supportive behavior may be at its first stage an adaptive role, especially when it occurs between related or associated individuals [47]. However, the benefits of this behavior appear less obvious when it is extended for long periods, suggesting that other factors such as attachment and social bond may participate in delaying abandonment [5].

In both observations, different types of vocalizations, named click train, burst pulses, bray call, and whistles were recorded. This evidence is consistent with other studies that reported different species of dolphins to vocalize consistently during epimeletic behavior, [9,17,48] although acoustic production in such contexts is still largely unknown. 

The detection of whistles, in particular, could be expected since they are known to play a fundamental role in bottlenose dolphins’ intraspecific communication and social activity [49,50]. However, only a couple of studies have characterized whistles emitted in epimeletic contexts, and they reported high emission rates [9,17], and increased duration and number of inflection points [9]. Longer and more complex whistles could convey information on the emotional state of the emitter [9]. Indeed bottlenose dolphins are known to change the acoustic parameters of their whistles depending on the contexts [51] and these variations could potentially act as stress indicators [16]. In the first event, a majority of modulated whistles (11/12 OWs) were recorded, that differed in duration and frequency parameters from the mean values observed in the Mediterranean sea [50,52]. All frequency parameters were higher in the present study with respect to the mean Mediterranean values, while duration and number of inflection points were slightly lower. Although being partially in contrast with Cheng et al. [9], these results are consistent with other studies that report higher vocalizations frequency in stressful conditions [16]. The variations in whistles’ acoustic parameters here reported could therefore be interpreted as the expression of contest specific information. However, it must be taken into account that the small sample size (OWs = 12) may have affected this result. In the same encounter, a signature whistle (SW_1) consistently repeated during the sighting was identified. This SW showed higher values of all frequency parameters, duration, and number of inflection points than OWs, possibly because higher-frequency vocalizations are commonly recorded in stress contexts [16] as well as longer and highly modulated whistles’ contour patterns [9]. Signature whistles can be used by an individual as distress calls, to seek aid from others in stressful situations [17,53]. When used as a distress call, a signature whistle is intensely repeated until sufficient aid is provided and could also appear quavering [6], suggesting that SW_1 here recorded was emitted by the main supporter to request aid. Another possible option is that the SW_1 was emitted by the putative mother to communicate with the calf since signature whistles are known to function as reunion calls for mother-calf pairs [54]. However, since we were not able to determine which individual emitted the SW_1, these hypotheses cannot be confirmed. The two observations highly differed in the number of whistles recorded, with fewer whistles detected in the second case. It may be hypothesized that the presence of a resident female actively supporting the putative mother (a resident individual as well) reduced her necessity of emitting her signature whistles, in contrast with the first case in which the escort individual was a putative male, occasionally showing possible aggressive behavior toward the presumed mother-calf pair. However, this hypothesis is merely speculative, since a wide number of other factors may have affected this result.

Interestingly, bray call elements in both sightings were detected, which have never been reported before in epimeletic contexts. Bray calls are poorly understood even now, and their function is still largely unknown, although they have been recorded a few times during social activities [55,56]. Their possible usage to convey important information in epimeletic contexts as well is still not determined. 

Click trains and burst pulses were also recorded during both encounters. Click trains are used for echolocation tasks [30] so their emission was likely related both to navigation and/or localization of the carcass in the water. To our knowledge, burst pulses have never been specifically reported in epimeletic contexts, but they are considered communication signals, emitted both during affiliative and agonistic social interactions, likely to convey information on the physical and emotional state of the emitter [30]. A similar function could therefore be presumed for the burst pulses detected in both our observations. 

As mentioned above, the carcasses were not recovered as to not disrupt the behavior of the putative mothers, so the cause of death remains uncertain. However, calf mortality is generally caused by water pollution, anthropogenic disturbance, or infanticide [9]. All these factors could have potentially caused the calves’ death as the area is strongly affected by anthropogenic pressure and receives a large quantity of pollutants carried by the Tiber river [21]; furthermore, infanticide has been frequently documented in bottlenose dolphins [40] and could have possibly occurred also in these cases. 

## 5. Conclusions

This anecdotal report describes two cases of nurturant behavior involving a dead calf and associated acoustic production in common bottlenose dolphins in the Mediterranean Sea. The two examples here reported confirm the occurrence of epimeletic behavior in the species and the second case corroborates the hypothesis that females play a predominant role as main supporters in such contexts. Furthermore, they provide a first description of the acoustic production and preliminary characterization of whistles emitted in contexts of epimeletic behavior involving a dead calf, which have never been documented before in bottlenose dolphins. The original information reported here may serve as an additional step toward a deeper comprehension of how cetaceans experience and interact with death-related contexts. Further research is needed to ascertain whether they can be aware of death and if their behavioral patterns could be considered as a non-human version of bereavement and grieving. The debate on this topic is still largely open since emotional responses to death have long been considered unique to humans. However, behavioral patterns comparable to grieving have been observed in terrestrial mammals [38] and, although they are not directly addressed as grieving, prolonged, extreme, and apparently maladaptive behaviors in response to the loss of a conspecific have been documented in cetaceans as well [5]. The lack of detailed data on the emotional states and the relationships between involved individuals makes it hard to classify such responses and highlights the necessity of reporting field observations for a deeper comprehension of this largely unknown topic. 

Finally, an important conservation issue should be addressed in the investigated area. Increased prey availability near river mouths is one of the possible drivers for the common bottlenose dolphin presence and abundance in the study site, which sustains a core nucleus of resident females with offspring [21]. However, anthropogenic disturbances and threats (vessel traffic, reduced prey availability caused by overfishing, plastic and chemical pollution, and habitat degradation, including noise) may impact distribution and individuals’ interactions, thus altering the social structure and influencing how the population responds to changes to its environment [57,58,59,60,61]. Future investigations should address the structure of the relationships between individuals as it is an essential aspect affecting animals’ responses to both human-related pressures and management actions [57,62].

## Figures and Tables

**Table 1:** Spectrogram setting used for each vocalization type in Raven Pro 1.6.

Vocalization	Spectrogram Parameters (Raven Pro 1.6)
Whistles	Hamming window, size 1024, DFT 1024, Overlap 50%, Hop size 512
Click trains/Burst Pulses	Hamming window, size 512, DFT 512, Overlap 50%, Hop size 256
Bray-call elements	Hann window, size 2048, DFT 2048, Overlap 50%, Hop size 1024

**Table 2:** Acoustic parameters extracted for good and high-quality whistles, click trains, and burst pulses.

**Whistles**	**Definition**
Minimum Frequency (Hz)	Frequency at the lower limit of the whistle
Maximum Frequency (Hz)	Frequency at the upper limit of the whistle
Frequency Range (Hz)	Maximum frequency-minimum frequency
Start Frequency (Hz)	Frequency at the beginning of the whistle
End Frequency (Hz)	Frequency at the end of the whistle
Duration (s)	Total duration calculated as: ending time-beginning time
Number of Inflection Points	Mathematic definition in sine function of a change from positive to negative or negative to positive slope
Harmonics (presence/absence)	Presence of multiples of the fundamental frequency
Step (presence/absence)	Abrupt discontinuity in frequency
Interruptions (presence/absence)	Abrupt discontinuity in time
**Click trains/Burst Pulses**	**Definition**
Number of pulses	Number of pulses composing the sound
Duration (s)	Time from the first click to the last click
Repetition Rate (pulse/second)	Number of pulses per second
Inter-click-interval (ms)	Inverse of the repetition rate

**Table 3:** Mean ± standard deviation of each acoustic parameter extracted from good and high-quality whistles.

Whistle Category	Maximum Frequency (Hz)	Minimum Frequency (Hz)	Frequency Range (Hz)	Start Frequency (Hz)	End Frequency (Hz)	Duration (s)	Inflection Points (N)
**OWs**	20507 ± 1794	7213 ± 1213	13294 ± 2472	9151 ± 2566	17802 ± 3759	0.98 ± 0.46	2.1 ± 1.7
**SW_1**	21969 ± 1075	6851 ± 989	15118 ± 1450	7579 ± 1667	18928 ± 1792	1.38 ± 0.06	3

## Data Availability

The data presented in this study are available to any qualified researcher on request from the corresponding author.
